# Key Developments in Translational Neuroscience: an Update

**DOI:** 10.4274/balkanmedj.2017.6.0002

**Published:** 2017-12-01

**Authors:** Drozdstoy St. Stoyanov

**Affiliations:** 1 Research Complex for Translational Neuroscience, Department of Psychiatry and Medical Psychology, Medical University Plovdiv, Plovdiv, Bulgaria

Contemporary scientific effort is no longer enterprise located within one single domain or matrix of knowledge. Mono-disciplinary investigations are already replaced with intensive and extensive cross-disciplinary connections. In most areas of natural sciences and medicine such connections are enabled by either reductive models (from more complex life systems to the basic models) or by established commensurability of the shared terms, notions and methods. This facilitates inter-domain translation within successful projects for coordination and consolidation of knowledge, e.g. quantum physics and chemistry, cognitive science among others.

Unfortunately this is not the case in psychiatry. Psychiatry operates with various disciplinary languages, adopted from neuroscience, social sciences and humanities. Those languages and methods are sometimes beyond comparison which is one major conceptual obstacle before their integration into current classifications and treatment guidelines.

The present section is dedicated to the issue of translation between neuroscience, psychology and psychopathology, which is addressed by a set of collaborative articles to demonstrate the state-of-the art and possible future agenda for cooperation. All articles include reference to original research of the authors who are considered to be crucial figures in their area of expertise.

Translational neuroscience has developed over the past few decades as innovative field to bridge knowledge across disciplines in medicine and beyond. By its original definition it was supposed to translate data/knowledge from fundamental neurosciences (such as neurobiology) which are essentially collected in animal models to explanation of human brain function in health and disease. However this agenda has been widened to the ongoing multidisciplinary framework, which involves many other types of translation, as presented in the review by Aragona (1). It provides us with insight into the many tacit meanings and implications behind the notion of translation as methodological enterprise.

Further empirical studies which relate functional magnetic resonance imaging to clinical assessment scales employed in psychiatry is delivered in the paper by Stoyanov et al. (2). There is provided also extensive background in terms of the translation of fMRI to other diagnostic methods, such as magnetic resonance spectroscopy, neuro-immunology, electrophysiology etc. The main rationale behind this approach is an attempt to deliver converging evidence from various disciplines to endorse the evaluation and treatment strategies in depression.

Also in the field of depression research, however from another view, Çalıyurt (3) paper brings together different bio-medical disciplines, intertwined in the pathophysiology and management of affective disorders. Chronobiology is considered in this perspective as a meta-discipline, which sets the background for both diagnostic assessment and treatment of mood disorders with light therapy and sleep deprivation.

The article by Hugdahl (4) summarizes an original model of the psychophysiological mechanism of auditory verbal hallucinations in schizophrenia. This review covers an extensive range of causal mechanisms: from neurochemistry to clinical phenomenology with an emphasis on innovative explanatory model of direct relevance to post-modern treatment techniques.

What is emerging from this collection of papers is an exciting horizon of crossing borders and disciplinary limitations underpinning the controversies in contemporary psychiatry. Neurobiology, physiology, functional neuroimaging and clinical domains happen to deliver sometimes incommensurable variables, methods and terminology. We attempt hereby to give an example of effective transdisciplinary and international cooperation. The papers from this section comprise contributions from such leading centers of excellence as the University of Bergen fMRI group, University of Basel Neuropsychiatry and Brain Imaging Group as well as from new and emerging centers of excellence in translational neuroscience as the Medical University of Plovdiv and the Trakya University in Edirne.

This is the intellectual product of synergy across domains of scientific inquiry in medicine and synergy in inter-institutional cooperation.
